# Concentrated Hypoxia-Preconditioned Adipose Mesenchymal Stem Cell-Conditioned Medium Improves Wounds Healing in Full-Thickness Skin Defect Model

**DOI:** 10.1155/2014/652713

**Published:** 2014-11-25

**Authors:** Biao Sun, Shilei Guo, Fei Xu, Bin Wang, Xiujuan Liu, Yuanyuan Zhang, Yan Xu

**Affiliations:** ^1^Department of Human Sports Science, Nanjing Institute of Physical Education, Nanjing 210014, China; ^2^Nanjing Regenerative Medicine Engineering and Technology Research Center, Nanjing 210046, China

## Abstract

In recent years, the bioactive factors were utilized in exercise and athletic skin injuries. In this research, the concentrated conditioned medium of hypoxia-preconditioned adipose mesenchymal stem cells, which is rich in bioactive factor, is applied in full-thickness skin defect model to evaluate the therapeutic efficacy. Adipose mesenchymal stem cells were harvested from the abdominal subcutaneous adipose tissues. The surface markers and the potential of differentiation were analyzed. The conditioned medium of hypoxia-preconditioned stem cells was collected and freeze-dried and then applied on the rat full-thickness skin defect model, and the healing time of each group was recorded. Haematoxylin and eosin staining of skin was assessed by microscope. The characteristics of adipose mesenchymal stem cells were similar to those of other mesenchymal stem cells. The concentration of protein in freeze-dried conditioned medium in 1 mL water was about 15 times higher than in the normal condition medium. In vivo, the concentrated hypoxia-preconditioned conditioned medium can reduce the wound size and accelerate the skin wound healing. The concentrated hypoxia-preconditioned adipose mesenchymal stem cell-conditioned medium has great effect on rat model of wound healing, and it would be an ideal agent for wound care in clinical application.

## 1. Introduction

It is well established that active life style is associated with improved quality of life. However, skin wound is one of the most common causes of inactivity (lack of movement) [1]. The skin wound healing is a complicated process requiring coordination of different tissues and cells, to ensure successful healing.

Adipose mesenchymal stem cells (AMSCs) have the ability to repair skin damage and promote wound healing. The requirements of cells culture limited the clinical application of stem cells. Furthermore, only a small percentage of cells will survive in damaged skin tissues. It is believed that the secretome of AMSCs plays an important role in skin wound healing [2, 3]. The conditioned medium of AMSCs (AMSCs-CM) accelerated wound closure with increased reepithelialization, cell infiltration, and angiogenesis [4]. Recent research showed that the low oxygen concentration could improve the effects of paracrine of the bone mesenchymal stem cells on murine skin wound healing [5].

In this study, we performed experiments using concentrated hypoxia-preconditioned AMSCs-CM (Conc. Hypo-AMSCs-CM) to evaluate the effects of concentration and nonconcentration of Hypo-AMSCs-CM on the rat's full-thickness skin defect model.

## 2. Materials and Methods

### 2.1. Isolation, Identification, and Characterization of ASC

The abdominal subcutaneous adipose tissue was collected from the female Sprague-Dawley rats (250–300 g). The adipose tissue was minced and digested with collagenase (0.12 U/mL, GIBCO, USA) at 37°C for 40 min under constant shaking. The cellular suspensions were passed through a 100 *μ*m cell strainer. After centrifugation (400 g for 10 min), the cellular pellet was resuspended in Dulbecco's Modified Eagle Medium/10% fetal bovine serum placed in concentration 2–4 × 10^4^ cells/cm^2^. The phenotype of AMSCs (passage 3) was assessed by indirect flow cytometry. The analysis (fluorescence-activated cell sorting, FACS) was performed using CD29, CD34, CD71, and CD90 as primary fluorescent antibodies and using IgG-FITC and IgG-PE as secondary antibodies. The negative control was cells without primary antibodies. To induce osteogenic and adipogenic differentiation, the medium was replaced with adipogenic or osteogenic differentiation medium, as described before (when AMSCs as passage 3 grew to approximately 90% confluence) [6]. The differentiated AMSCs were stained with Oil Red O for analysis of lipid droplet in adipogenic induction or Alizarin Red for calcium in osteogenic induction.

### 2.2. Establishment of Hypoxia Microenvironment

As described previously, cells were cultured in the sealed box with an Anaeropack, a disposable oxygen-absorbed and CO_2_ generator, for 24 h at 37°C [7]. The Anaeropack starts to absorb oxygen within 1 min; oxygen tension inside the box drops to 1 mm Hg within 1 h. The final concentration of oxygen was less than 1%, and the concentration of carbon dioxide was about 20%.

### 2.3. Lyophilization of AMSCs-CM

The AMSCs culture dishes were washed three times with PBS and cultured overnight in culture medium consisting of DMEM/F-12, 0.4% rat serum. After 24 hours of incubation, the cultured medium was collected and then the fresh low-serum medium was added. The cultured medium of AMSCs was filtered using a 0.22 *μ*m filter and then centrifuged at Amicon-Ultra-15 mL (MWCO 3 kD, Millipore) at 4°C 4000 g for 30 minutes. The medium was concentrated about 5 times using ultrafiltration membrane of 3.5 kD with polyethylene glycol at 4°C. The conditioned medium was concentrated about 15 times as described above. Freezing-dried powder was prepared in the sterilized penicillin bottle using the Lyophilizer (Boyikang Corp., Beijing, China). The concentration of protein in conditioned medium was measured by NanoDrop 2000 (Thermo, MA, USA).

### 2.4. Creation of Full-Thickness Skin Defect Model and Conditioned Medium Transplantation

All animal experiments were conducted with the approval of Nanjing Medical University Ethics Committee and were performed in accordance with the Guide for the Care and Use of Laboratory Animals published by the US National Institutes of Health (NIH Publication number 86-23, revised 1996). The full-thickness skin defect model used in this study is identical to the model previously described [8]. Thirty adult female Sprague-Dawley rats, weighing 200 g, were used. After induction of general anesthesia, the dorsal regions of the rats were shaved. Then the dorsal skin was disinfected with iodine tincture and 75% alcohol. Full-thickness skin incisions, 30 mm in diameter, were made on the back of each rat (Figure 2(a)(1)). Thirty rats were randomly divided into 3 groups: (1) concentrated hypoxia-preconditioned adipose mesenchymal stem cells-conditioned medium group (Conc. Hypo-AMSCs-CM group); (2) ASC-conditioned medium group (Hypo-AMSCs-CM group); (3) control group (0.4% rat serum medium). The location of skin injury in all experimental groups was covered with concentrated and normal Hypo-AMSCs-CM, which contains 1% hyaluronic acid (HA). 1% hyaluronic acid with rat serum medium was only applied to the control group. All medium was attached to the surface of wound completely and fixed on the skin with sterile transparent patches. Digital pictures were taken to visualize the wounds.

### 2.5. Statistical Analysis

The results are expressed as mean ± standard deviation (SD). Statistical analysis was performed using analysis of variance (ANOVA) test by SPSS17.0, as appropriate. The *t*-test analysis was applied for measuring the data, and the regression analysis was used for the relationship between the time and the percentage of wound healing. *P* < 0.05 was considered to be statistically significant.

## 3. Results

### 3.1. Characterization of Adipose Stem Cell and Conditioned Medium

**Figure 1:**
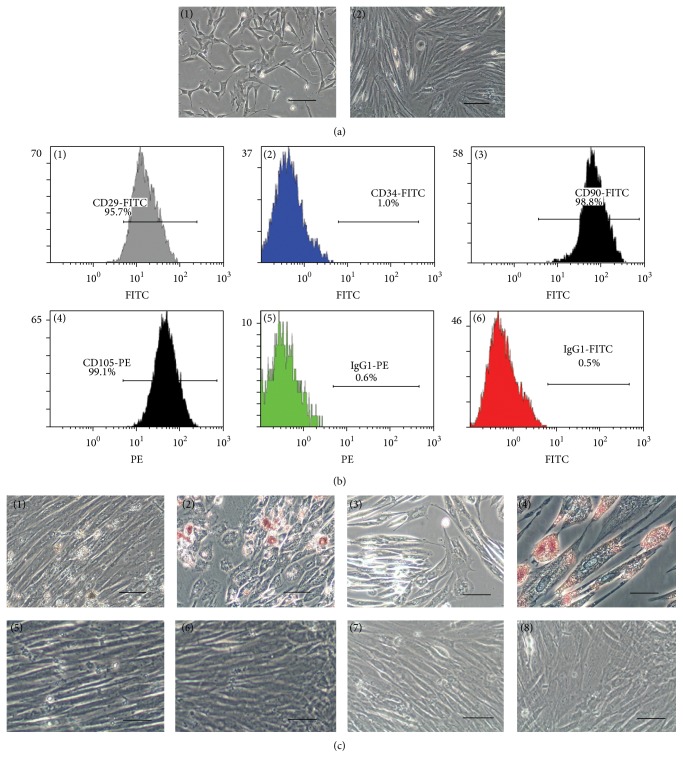
Characterization of adipose mesenchymal stem cells. The primary adipose mesenchymal stem cells were cultured in 7 days ((a)(1)) and exhibited a typical fibroblast-like morphology at passage 3 after 27 days ((a)(2)). For flow cytometry analysis, the results confirmed that the cells express CD29 (95.7%, (b)(1)), CD90 (98.8%, (b)(3)), and CD105 (99.1%, (b)(4)), but not CD34 (1.0%, (b)(2)). PE (0.6%, (b)(5)) and FITC (0.5%, (b)(6)) were performed as negative control. Oil Red O stain of lipid after differentiation for 28 days (before staining, (c)(1); after staining, (c)(2)), Alizarin Red staining of calcium after differentiation (before staining, (c)(3); after staining, (c)(4)), the Oil Red O stain of noninduced cells (before staining, (c)(5); after staining, (c)(6)), and the Alizarin Red stain of noninduced cells (before staining, (c)(7); after staining, (c)(8)). Scale bar = 100 *μ*m.

**Figure 2:**
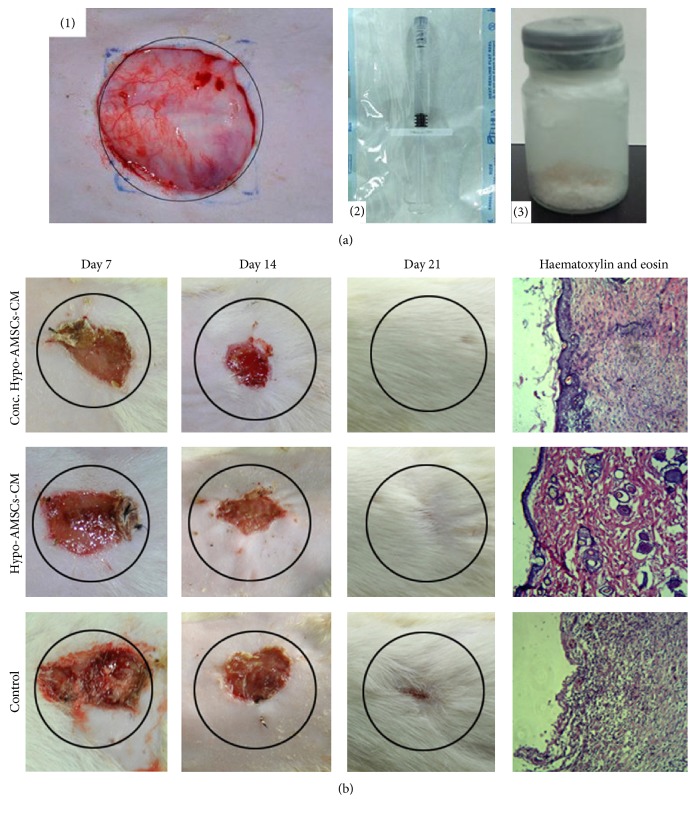
Transplantation of Conc. Hypo-AMSCs-CM enhanced the wound healing. The full-thickness skin incisions were about 30 mm in diameter ((a)(1)). The freezing-dried powder of Hypo-AMSCs-CM was collected in the sterilized penicillin bottle ((a)(3)) and diluted in the 1% hyaluronic acid ((a)(2)) and attached to the surface of wound completely. Representative images of the wound at special time points (7, 14, and 21 days) in each group were showed (b). For H&E stain, biopsies from center of wound in Conc. Hypo-AMSCs-CM showed well organized epidermis and thick cuticular layer (b).

The average number of adipose mesenchymal stem cells isolated was 1.4 ± 0.5 × 10^6^/mL from abdominal fat. Primary AMSCs were cultured for 7 days (Figure 1(a)(1)) and showed a typical fibroblast-like morphology at passage 3 after 27 days (Figure 1(a)(2)). The result of flow cytometry confirmed that AMSCs expressed CD29 (Figure 1(b)(1)), CD90 (Figure 1(b)(3)), and CD105 (Figure 1(b)(4)), but not CD34 (Figure 1(b)(2)). Oil Red O staining showed the lipid vacuoles appearance in cells (Figure 1(c)(2)) and Alizarin Red staining obviously revealed calcium in cells, as observed from red staining (Figure 1(c)(4)). Meanwhile, the Oil Red O staining of noninduced cells (before staining, (c)(5); after staining, (c)(6)) and the Alizarin Red staining of noninduced cells were also shown in Figure 1 (before staining, (c)(7); after staining, (c)(8)). The Hypo-AMSCs-CM was collected and lyophilized in the sterilized penicillin bottle. In application, the Conc. Hypo-AMSCs-CM was diluted with 1 mL sterilized water or hyaluronic acid (Figure 2(a)(2)). The protein concentration of Hypo-AMSCs-CM is 0.389 ± 0.04 mg/mL, the Conc. Hypo-AMSCs-CM is 5.989 ± 0.07 mg/mL, and the 0.4% rat serum medium is 0.350 ± 0.04 mg/mL.

### 3.2. Enhanced Wound Healing by Conditioned Medium

**Figure 3:**
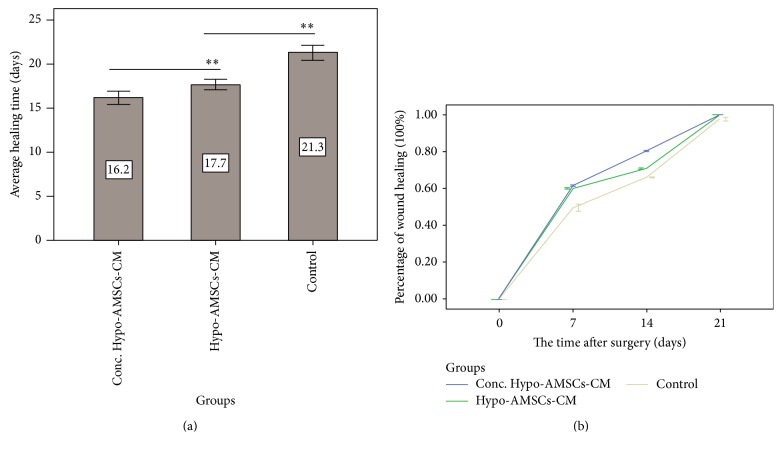
The average healing time and percentage of wound healing in each group. The average healing time of Conc. Hypo-AMSCs-CM group (10 rats) is 16.2 ± 0.98 days, Hypo-AMSCs-CM group (10 rats) is 17.7 ± 0.78 days, and the control group (10 rats) is 21.3 ± 1.10 days (a). There was significant difference between the Conc. Hypo-AMSCs-CM and Hypo-AMSCs-CM groups (*P* = 0.002) and between Hypo-AMSCs-CM and control groups (*P* < 0.001). Percentage of wound healing of groups on 7, 14, and 21 days after surgery (b). The Conc. Hypo-AMSCs-CM treatment improved wound healing compared with the Hypo-AMSCs-CM at 7 (*P* < 0.001) and 14 (*P* < 0.001) days (Figures 2(b) and 3(b)). The Hypo-AMSCs-CM group's wound healing was also significantly different from that of the control group at 7 (*P* < 0.001), 14 (*P* < 0.001), and 21 days (*P* = 0.0005) (Figures 2(b) and 3(b)) too.

The average healing time of the three groups was measured, of which the Conc. Hypo-AMSCs-CM group was 16.2 ± 0.98 days, Hypo-AMSCs-CM group was 17.7 ± 0.78 days, and medium control group was 21.3 ± 1.10 days (Figure 3(a)). There were significant differences between the Conc. Hypo-AMSCs-CM and Hypo-AMSCs-CM groups (*P* = 0.002) and between Hypo-AMSCs-CM and control groups (*P* < 0.001). Percentage of wound healing was evaluated by measurement of “healing area/initial wound area × 100%” on 7, 14, and 21 days after treatment in three groups (Figure 3(b)). In the regression analysis, the equation of relationship between the time and the percentage of wound healing in Conc. Hypo-AMSCs-CM group was *y* = 0.0455*x* + 0.127 (*R* ^2^ = 0.9043, *P* < 0.01); that in Hypo-AMSCs-CM group was *y* = 0.0444*x* + 0.1108 (*R* ^2^ = 0.9122, *P* < 0.01); and that in the control group was *y* = 0.0442*x* + 0.0688 (*R* ^2^ = 0.9589, *P* < 0.01). There were no differences between the Conc. Hypo-AMSCs-CM and Hypo-AMSCs-CM groups (*P* = 0.299). The Conc. Hypo-AMSCs-CM treatment improved wound healing when compared with that of the Hypo-AMSCs-CM group at 7 (*P* < 0.001) and 14 (*P* < 0.001) days (Figures 2(b) and 3(b)). The Hypo-AMSCs-CM group's wound healing was also significantly different from that of the control groups at 7 (*P* < 0.001), 14 (*P* < 0.001), and 21 days (*P* = 0.0005) (Figures 2(b) and 3(b)). For hematoxylin and eosin stain, biopsies of three groups were harvested from center of wound at 21 days after wound. Wound closure after treatment with Conc. Hypo-AMSCs-CM showed well organized epidermis, thick cuticular layer, and increased collagen content (Figure 2(b)), whereas wound areas treated with Hypo-AMSCs-CM showed immature epidermal regeneration, weak cuticular layer, and less collagen content (Figure 2(b)). Collectively, these data showed that Conc. Hypo-AMSCs-CM treatment has better effects on wound repair than Hypo-AMSCs-CM treatment.

## 4. Discussion

It has been shown that AMSCs can differentiate into adipogenic and osteogenic lineages in the particular medium [9]. In this study, AMSCs showed the same immune-phenotypes as described previously [6, 10]. They expressed the mesenchymal stem cell markers CD29, CD90, and CD105, but not hematopoietic marker CD34.

The applications of culture medium of other cells have also been widely used in wound healing. We searched the MEDLINE and EMBASE databases, using the “conditioned medium” and “skin wound” as search terms. All eligible studies were parallel-controlled trials about conditioned medium of stem cell in skin wound healing (see Supp. l in Supplementary Material available online at http://dx.doi.org/10.1155/2014/652713). We first freeze-dried the conditioned medium of AMSCs, so the multiple concentration of conditioned medium could be made for clinical application.

However, the umbilical cord Wharton's jelly cells [11] and bone marrow mesenchymal cells [12] were used frequently, and the adipose mesenchymal stem cells were selected in this research not only because of extensive sources, but also because of the fact that the fat tissue plays an important role in reepithelialization of damaged tissue and reconstruction of skin appendage. Subcutaneous adipose cells, which demonstrate the dynamic regeneration parallel to the activation of skin stem cell, are necessary and sufficient to drive follicular stem cell activation and support lanugo growth [13]. The Hypo-AMSCs-CM has been shown to promote hair growth in C(3)H/NeH mice [14].

AMSCs were located in perivascular niches, close to vascular structure in fat. In vivo, the oxygen concentration of fat tissue is 2%–8% [15]. The lower oxygen environment is an important factor for maintaining the self-renewal and plastic characterization of AMSCs. In vitro, the oxygen concentration is considered a driver of cell function, and it helps maintain stem cell pluripotency, induces angiogenesis, and regulates the signaling of stem cell [16]. The Hypo-AMSCs-CM could enhance the wound healing via angiogenesis, increasing the collagen 1 expression, migration of fibroblasts, vascular endothelial cell and keratinocytes, and recruiting the circulating stem cells. Therefore, response of hypoxia-preconditioned AMSCs may be invaluable for the development of novel wound healing and skin regeneration strategies [17].

Skin is the first line of body's defense in the protection of foreign invasion, as the skin is exposed to pathogens in the macroenvironment. The unexpected skin injury may affect circulative exercise training, thus disrupting normal training rhythm. Serious injury could potentially end athlete's whole sports career. According to the skin wound severity, the healing takes around 2 weeks generally. Many treatments have been proven to accelerate the healing, such as tissue engineering [18], stem cell [19], PRP [20], and gene therapy [21]. However, the cost and safety are major challenges. The results from this study showed that there are no statistical differences between the concentrated and nonconcentrated groups. The process of concentration may be important for wound healing because of difference in day 7 and day 14. Further, the expanded sample size is necessary. Additionally, the Hypo-AMSCs-CM has several advantages in skin wound healing: (1) being without nucleic acid and suitable for allogeneic application, (2) terminal sterilization by filtration, (3) easiness in producing and maintaining, and (4) a variety of concentrations of AMSCs-CM that could be obtained from freezing-dried powder.

## 5. Conclusion

The Hypo-AMSCs-CM could be powdered through the concentrated and lyophilized process, and the high concentrated Hypo-AMSCs-CM, made by the powder, may be more effective in the skin wound treatment of the rat model.

## Supplementary Material

Supplementary Table 1: The summary of application of culture medium in wound healing. The sources of culture medium is most from adipose-derived stem cells, bone marrow mesenchymal stem cells and Wharton's jelly mesenchymal stem cells. And the hypoxia is the most common preconditioned way in cell culture. The growth factor in culture medium promote wound healing more rapid compare with placebo and stem cells, and hypoxia preconditioned could enhance the wound healing function of culture medium.

## References

[1] Godwin E. E., Young N. J., Dudhia J., Beamish I. C., Smith R. K. W. (2012). Implantation of bone marrow-derived mesenchymal stem cells demonstrates improved outcome in horses with overstrain injury of the superficial digital flexor tendon. *Equine Veterinary Journal*.

[2] Modo M., Rezaie P., Heuschling P., Patel S., Male D. K., Hodges H. (2002). Transplantation of neural stem cells in a rat model of stroke: assessment of short-term graft survival and acute host immunological response. *Brain Research*.

[3] Salgado A. J. B. O. G., Reis R. L. G., Sousa N. J. C., Gimble J. M. (2010). Adipose tissue derived stem cells secretome: soluble factors and their roles in regenerative medicine. *Current Stem Cell Research and Therapy*.

[4] Yew T. L., Hung Y. T., Li H. Y. (2011). Enhancement of wound healing by human multipotent stromal cell conditioned medium: the paracrine factors and p38 MAPK activation. *Cell Transplantation*.

[5] Chen L., Xu Y., Zhao J. (2014). Conditioned medium from hypoxic bone marrow-derived mesenchymal stem cells enhances wound healing in mice. *PLoS ONE*.

[6] Zuk P. A., Zhu M., Ashjian P. (2002). Human adipose tissue is a source of multipotent stem cells. *Molecular Biology of the Cell*.

[7] Sato H., Sato M., Kanai H. (2005). Mitochondrial reactive oxygen species and c-Src play a critical role in hypoxic response in vascular smooth muscle cells. *Cardiovascular Research*.

[8] Xiong B., Gao J.-H., Chen H.-D. (2009). Establishment of a rat model of bone marrow mesenchymal stem cell transplantation for repairing full-thickness skin defect. *Nan Fang Yi Ke Da XueXueBao*.

[9] Zuk P. A., Zhu M., Mizuno H. (2001). Multilineage cells from human adipose tissue: implications for cell-based therapies. *Tissue Engineering*.

[10] Moon M. H., Kim S. Y., Kim Y. J., et al (2006). Human adipose tissue-derived mesenchymal stem cells improve postnatal neovascularization in a mouse model of hindlimb ischemia. *Cellular Physiology and Biochemistry*.

[11] Fong C.-Y., Tam K., Cheyyatraivendran S. (2014). Human Wharton's jelly stem cells and its conditioned medium enhance healing of excisional and diabetic wounds. *Journal of Cellular Biochemistry*.

[12] Chen L., Tredget E. E., Wu P. Y. G., Wu Y., Wu Y. (2008). Paracrine factors of mesenchymal stem cells recruit macrophages and endothelial lineage cells and enhance wound healing. *PLoS ONE*.

[13] Festa E., Fretz J., Berry R. (2011). Adipocyte lineage cells contribute to the skin stem cell niche to drive hair cycling. *Cell*.

[14] Park B. S., Kim W. S., Choi J. S. (2010). Hair growth stimulated by conditioned medium of adipose-derived stem cells is enhanced by hypoxia: Evidence of increased growth factor secretion. *Biomedical Research*.

[15] Pasarica M., Sereda O. R., Redman L. M. (2009). Reduced adipose tissue oxygenation in human obesity evidence for rarefaction, macrophage chemotaxis, and inflammation without an angiogenic response. *Diabetes*.

[16] Lin Q., Kim Y., Alarcon R. M., Yun Z. (2008). Oxygen and cell fate decisions. *Gene Regulation and Systems Biology*.

[17] Chung H.-M., Won C.-H., Sung J.-H. (2009). Responses of adipose-derived stem cells during hypoxia: enhanced skin-regenerative potential. *Expert Opinion on Biological Therapy*.

[18] Chen M., Przyborowski M., Berthiaume F. (2009). Stem cells for skin tissue engineering and wound healing. *Critical Reviews in Biomedical Engineering*.

[19] Lee S. H., Jin S. Y., Song J. S., Seo K. K., Cho K. H. (2012). Paracrine effects of adipose-derived stem cells on keratinocytes and dermal fibroblasts. *Annals of Dermatology*.

[20] Park H. B., Yang J. H., Chung K. H. (2011). Characterization of the cytokine profile of platelet rich plasma (PRP) and PRP-induced cell proliferation and migration: upregulation of matrix metalloproteinase-1 and -9 in HaCaT cells. *Korean Journal of Hematology*.

[21] Penn J. W., Grobbelaar A. O., Rolfe K. J. (2012). The role of the TGF-beta family in wound healing, burns and scarring: a review. *International Journal of Burns and Trauma*.

