# Supplementary material for: Concentrated Hypoxia-Preconditioned Adipose Mesenchymal Stem Cell-Conditioned Medium Improves Wounds Healing in Full-Thickness Skin Defect Model
Source: Int Sch Res Notices. 2014 Nov 25;2014:652713. doi: 10.1155/2014/652713 (PMC4897251; doi:10.1155/2014/652713)
Supplement: Supplementary file 1 — Supplementary Table 1: The summary of application of culture medium in wound healing. The sources of culture medium is most from adipose-derived stem cells, bone marrow mesenchymal stem cells and Wharton's jelly mesenchymal stem cells. And the hypoxia is the most common preconditioned way in cell culture. The growth factor in culture medium promote wound healing more rapid compare with placebo and stem cells, and hypoxia preconditioned could enhance the wound healing function of culture medium. [file 652713.f1.pdf]

Table 1. Characteristic of the included studies

Author (year)	Research type	the kind of cells	Precontioned	Model	conditioned medium group	other group	Assessment in vivo	Conclusion
Tam K(2014)[11]	parallel controlled design	Wharton`s jelly of human umbilical cords mesenchymal stem cells(hWJSCs)	None	Excisional wounds in mice; Diabetic wounds in immunodeficient mice	hWJSCs-CM(conditioned medium)+AV/PCL(aloe vera-polycaprolactone); CCD(Human foreskin fibroblasts)-CM+AV/PCL	hWJSCs+AV/PCL; CCD+AV/PCL; PBS+AV/PCL; untreated	rapid wound closure; wound closure rates; histological examination; TaqMan qRT-PCR	hWJSCs with nanoscaffold may be a idea wound dressings for slow healing and hard-to-heal wounds
Jun EK(2014)[12]	parallel controlled design	Human amniotic fluid-derived mesenchymal stem cells(AF-MSCs)	Hypoxia	excisional wounds splinting model	AF-MSCs-hypoxia CM; AF-MSCs-normal CM	Vehicle medium(DMEM/F12)	General observation; degree of wound closure; H&E staining; Immunohistochemistry	AF-MSCs-hypoxia CM improves the wound healing through fibronectin-enhanced cell migration and TGF-β/SMAD2 and PI3K/AKT signal pathways
Fong CY(2014)[13]	parallel controlled design	Wharton`s jelly of human umbilical cords mesenchymal stem cells(hWJSCs)	None	excisional wounds in mice; diabetic wounds in immunodeficient mice	hWJSCs-CM; CCD(Human foreskin fibroblasts)-CM	hWJSCs; CCD; unconditioned medium	General observation; percentage healing rates; Immunohistochemistry; molecular analysis	hWJSCs enhance healing of excisional and diabetic wounds via differentiation and secretion
Chen L(2014)[5]	parallel controlled design	Bone marrow-derived mesenchymal stem cells(BM-MSC)	Hypoxia	full-thickness excisional wounds in nude mice	BM-MSC normal CM; BM-MSC hypoxia CM	Vehicle control	Immunohistochemical; Immunofluorescence	Hypoxic BM-MSCs and their secreted products might be enhance tissue repair subcutaneous injury
Arno AI(2014)[14]	parallel controlled design	Human Wharton`s jelly-derived Mesenchymal stem cells(WJ-MSCs)	None	Full-thickness skin excisional wounds in BALB/c mice	WJ-MSC-CM with Matrigel	Non-conditioned medium with Matrigel	Wound healing rates; Immunohistochemisty(Brdu)	Human WJ-MSCs promote wound healing by paracrine signaling in culture conditions in vivo model
Zhou BR(2013)[15]	parallel controlled design	Human adipose-derived stem cells(ADSCs)	None	Fractional carbon dioxide laser resurfacing(FxCR)on human skin	ADSCs-CM	Dulbecco`s modified Eagle`s Medium(DMEM)	Dermatological changes(erythema, melanin, TEWL, elasticity); Histopathological analysis(H&E, Masson-Trichrome, Gomori`s aldehyde fuchsin staining)	Allogenic ADSC-CM could be an effective method for enhancing wound healing and reducing transient unwanted adverse effects after FxCR skin rejuvenation
Tamari M(2013)[16]	parallel controlled design	Bone marrow-derived mesenchymal stem cells(BM-MSC)	None	Excisional wound-splinting mouse model	BM-MSC-CM	BM-MSC; Phosphate-buffered saline(PBS)(control)	Macroscopical and histological observation	MSC-CM contains growth factor derived from stem cells, is able to accelerate wound healing as well as stem cell transplantation
Zimber MP(2012)[17]	parallel controlled design	Human cells(BM-MSCs?)	Hypoxia	Human postlaser wounds	Concentration of human Cells conditioned medium(hCCM)(×0.1, ×1, ×10)	Gel without hCCM	Erythema; edema; dryness; peeling; transepidermal water loss	The utility of ×10 concentration hCCM appears to promote more rapid, scarless wound healing after resurfacing procedures and more normal skin recovery

Mishra PJ(2012)[18]	parallel controlled design	Bone marrow-derived human mesenchymal stem cells(BMD-hMSCs)	None	Excisional wounds in NOD/SCID and nude mice	Conditioned medium concentrate from hMSC(Hmsc(CMC))	Saline (control) group	Healing time; Immunohistochemistry	Cell-free derivatives of human MSCs are useful for wound healing purposes
Yew TL(2011)[4]	parallel controlled design	Bone marrow-derived mesenchymal stem cells(BM-MSCs)	None	Excisional wound splinting model in BALB/c mice	Conditioned medium from MSCs; Conditioned medium from MSCs+anti IL-6	α-minimal essential medium(α-MEM); α-MEM+IL-6	The percentage of wound closure; Histologic examination	MSCs promote wound healing through releasing a repertoire of paracrine factors via activation of p38 MAPK
Lee MJ(2011)[19]	parallel controlled design	Human embryonic stem cell-derived endothelial precursor cells(hESC-EPC)	None	Excisional wound model in BALB/c nude mice	hESC-EPC conditioned medium;Cord blood-EPC conditioned medium	Control vehicle medium	Wound closure time; the percentage of wound closure; immunohistochemical analysis	The spectrum of cytokines released by hESC-EPC are functionally involved in the wound healing process
Heo SC(2011)[20]	parallel controlled design	Human adipose-derived stem cells(ASCs)	Tumor necrosis factor(TNF)-α treated	Cutaneous wound healing model in Sprague-Dawley rats	TNF-α conditioned medium; control contioned medium	PBS(control)	Immunocytochemistry and histological analysis	TNF-α-activated ASCs accelerate cutaneous wound healing through paracrine mechanisms involving IL-6 and IL-8
Yoon BS(2010)[21]	parallel controlled design	Amniotic fluid-derived mesenchymal stem cells(AF-MSCs)	None	Excisional wound model in mice	AF-MSCs-conditioned medium	Control medium	The percentage of wound closure; histologic examination; immunochemistry	AF-MSCs secreted high levels of factors, which could enhance wound healing, may represent a novel therapy to improve the effectiveness of tissue repair
Cho JW(2010)[22]	parallel controlled design	Adipose-derived stem cells(ADSCs)	Tumor growth factor(TGF)-β 1 treated	Excisional wound model in hairless mice	TGF-β1-treated ADSCs-conditioned medium; ADSCs-conditioned medium(control)	-	Wound reducing size; histological analysis(?Not in result)	ADSCs-CM play important roles on promotion of wound healing in skin through up-regulation of type 1 collagen, MMP-1 expression, migration and proliferation of fibroblasts
Templin C(2009)[23]	parallel controlled design	Immortalization of murine haematopoietic progenitor cells(Dkmix cells)	None	Full-thickness excisional wound model in C57BL/6 mice	Dkmix cells` conditioned medium	Dkmix cells; PBS	The precentage of wound closure; Histological examination	Immortalized haematopoietic progenitor cells(Dkmix cells) significantly improve dermal wound healing by paracrine effects
Lee EY(2009)[24]	parallel controlled design	Adipose-derived stem cells(ADSCs)	Hypoxia	Full-thickness wounds in female hairless mice	Hypoxia-ADSCs conditioned medium; Normal-ADSCs conditioned medium	-	Remaining wound area	Hypoxia increases the proliferation of ADSCs and enhance the wound healing function of ADSCs,at least partly, by up-regulating the secretion of VEGF and bFGF
Kellar RS(2009)[25]	parallel controlled design	Neonatal fibroblasts	Hypoxia	Ablative Fractional Erbium Laser in the peri-oral and peri-ocular; nonablative Fractional Erbium Laser on the remainder of face	Hypoxic conditioned culture medium(HCCM) of neonatal fibroblasts(ReGenica™)	Vehicle gel formulation(control)	The amount of erythema(clinical evaluation, bioinstrumental assessment)	This HCCM product greatly improved in erythema and re-epithelization of the peri-oral and peri-ocular regions.
Chen L(2008)[26]	parallel controlled design	Bone marrow derived mesenchymal stem cells(BM-MSCs)	None	Excisional wound splinting model in BALB/c mice	Fibroblast-conditioned medium;MSC-conditioned medium	Pre-conditioned medium	The percentage of wound closure; Immunostaining and confocal microscopy	BM-MSCs released high levels of cytokines and chemokines which could enhance normal wound healing.
